# A mathematical modeling perspective on the advantages of single receptor type convergence in the olfactory glomeruli

**DOI:** 10.1186/1471-2202-14-S1-P428

**Published:** 2013-07-08

**Authors:** Lakshmi Chandrasekaran, C Ron Yu

**Affiliations:** 1 Stowers Institute for Medical Research, 1000 East 50th Street, Kansas City, MO 64110, USA; 2 Department of Anatomy and Cell Biology, University of Kansas Medical Center, Kansas City, KS 66160, USA

## 

Olfactory circuits in both insects and rodents are wired according to a pattern of convergence in which axons from olfactory sensory neurons expressing a single receptor type (SRT) project into the same glomerulus. This permits the representation of individual olfactory receptors in a topographical map in the brain ([1], [2], [3]). The functional advantage offered by a SRT convergence pattern is an open question. We analyze a simple mathematical model based on the anatomy of the olfactory bulb. The model contains two interconnected layers of glomeruli and mitral cells. We use the mathematical model to qualitatively explore the impact on odor coding and discrimination by changing the projection pattern from SRT to multiple receptor type (MRT) input patterns. We predict that for odors activating similar patterns, the MRT network is not able to separate response patterns as well as the SRT network. The mathematical prediction is in good qualitative agreement with experimental findings. Our predictions also indicate that sparseness of input patterns and overall network size are key parameters in determining how well the SRT network performs in discrimination of odors. Future work will focus on verification of these additional predictions through biological experiments and also investigating the role of more realistic network models in influencing odor discrimination abilities of the SRT case.
